# Supplementary material for: A genome-wide functional genomics approach uncovers genetic determinants of immune phenotypes in type 1 diabetes
Source: eLife. 2022 May 31;11:e73709. doi: 10.7554/eLife.73709 (PMC9205632; doi:10.7554/eLife.73709)
Supplement: Transparent reporting form [file elife-73709-transrepform1.docx]

***eLife’s* transparent reporting form**

We encourage authors to provide detailed information *within their submission* to facilitate the interpretation and replication of experiments. Authors can upload supporting documentation to indicate the use of appropriate reporting guidelines for health-related research (see [EQUATOR Network](http://www.equator-network.org/%20)), life science research (see the [BioSharing Information Resource](https://biosharing.org/)), or the [ARRIVE guidelines](http://www.plosbiology.org/article/info:doi/10.1371/journal.pbio.1000412) for reporting work involving animal research. Where applicable, authors should refer to any relevant reporting standards documents in this form.

If you have any questions, please consult our Journal Policies and/or contact us: [editorial@elifesciences.org](mailto:editorial@elifesciences.org).

**Sample-size estimation**

- You should state whether an appropriate sample size was computed when the study was being designed
- You should state the statistical method of sample size computation and any required assumptions
- If no explicit power analysis was used, you should describe how you decided what sample (replicate) size (number) to use

Please outline where this information can be found within the submission (e.g., sections or figure legends), or explain why this information doesn’t apply to your submission:

The total number included in the study has been described in the introduction and figure 1. The samples included in the genetic association analysis were carefully described in the method section (under the subtitle **Genotyping, quality control and imputation**)

**Replicates**

- You should report how often each experiment was performed
- You should include a definition of biological versus technical replication
- The data obtained should be provided and sufficient information should be provided to indicate the number of independent biological and/or technical replicates
- If you encountered any outliers, you should describe how these were handled
- Criteria for exclusion/inclusion of data should be clearly stated
- High-throughput sequence data should be uploaded before submission, with a private link for reviewers provided (these are available from both GEO and ArrayExpress)

Please outline where this information can be found within the submission (e.g., sections or figure legends), or explain why this information doesn’t apply to your submission:

Biological replication in reported cohort and the handling of outliers and possible contaminations were described in **Material and Methods** section.

We didn’t have an independent cohort as replication which is a limitation of our study. We have carefully discussed this limitation in the **Discussion** section of the manuscript. This is because that the population data (especially patients’ population data) are not always easy to acquire. As we know, this is the first systematic study illustrating genetic effects on cytokine production under stimulation in type 1 diabetes patients. Furthermore, comparison between our findings and in other relevant reports and functional exploration have provided some indirect evidence showing that our data and results are reliable.

**Statistical reporting**

- Statistical analysis methods should be described and justified
- Raw data should be presented in figures whenever informative to do so (typically when N per group is less than 10)
- For each experiment, you should identify the statistical tests used, exact values of N, definitions of center, methods of multiple test correction, and dispersion and precision measures (e.g., mean, median, SD, SEM, confidence intervals; and, for the major substantive results, a measure of effect size (e.g., Pearson's r, Cohen's d)
- Report exact p-values wherever possible alongside the summary statistics and 95% confidence intervals. These should be reported for all key questions and not only when the p-value is less than 0.05.

Please outline where this information can be found within the submission (e.g., sections or figure legends), or explain why this information doesn’t apply to your submission:

All statistically methods and necessary details have been carefully described in the method section and/or with relevant references.

(For large datasets, or papers with a very large number of statistical tests, you may upload a single table file with tests, Ns, etc., with reference to sections in the manuscript.)

**Group allocation**

- Indicate how samples were allocated into experimental groups (in the case of clinical studies, please specify allocation to treatment method); if randomization was used, please also state if restricted randomization was applied
- Indicate if masking was used during group allocation, data collection and/or data analysis

Please outline where this information can be found within the submission (e.g., sections or figure legends), or explain why this information doesn’t apply to your submission:

All statistically methods and necessary details have been carefully described in the method section and/or with relevant references.

**Additional data files (“source data”)**

- We encourage you to upload relevant additional data files, such as numerical data that are represented as a graph in a figure, or as a summary table
- Where provided, these should be in the most useful format, and they can be uploaded as “Source data” files linked to a main figure or table
- Include model definition files including the full list of parameters used
- Include code used for data analysis (e.g., R, MatLab)
- Avoid stating that data files are “available upon request”

Please indicate the figures or tables for which source data files have been provided:

Results reported in the manuscript has been summarised in supplementary tables. Codes for generating all results could be found at github (<https://github.com/Chuxj/Gf_of_ip_in_T1D>). Raw immune phenotype data (cell proportion and cytokine production in 300DM) and summary statistics could be found at Dryad (https://doi.org/10.5061/dryad.4f4qrfjd0). Genetics and donor information that could compromise research participant privacy are only vailable upon request to the corresponding authors (http://hfgp.bbmri.nl).
